# Integrative pan-cancer analysis and clinical characterization of the N7-methylguanosine (m7G) RNA modification regulators in human cancers

**DOI:** 10.3389/fgene.2022.998147

**Published:** 2022-09-26

**Authors:** Chun-Ming He, Xin-Di Zhang, Song-Xin Zhu, Jia-Jie Zheng, Yu-Ming Wang, Qing Wang, Hang Yin, Yu-Jie Fu, Song Xue, Jian Tang, Xiao-Jing Zhao

**Affiliations:** ^1^ Department of Thoracic Surgery, Renji Hospital, Shanghai Jiaotong University School of Medicine, Shanghai, China; ^2^ Department of Cardiovascular Surgery, Renji Hospital, Shanghai Jiaotong University School of Medicine, Shanghai, China

**Keywords:** pan-cancer analysis, m7G regulators, tumor microenevironment, survival, immune score, drug sensitivity

## Abstract

**Background:** RNA modification is one of the epigenetic mechanisms that regulates post-transcriptional gene expression, and abnormal RNA modifications have been reported to play important roles in tumorigenesis. N7-methylguanosine (m7G) is an essential modification at the 5′ cap of human mRNA. However, a systematic and pan-cancer analysis of the clinical relevance of m7G related regulatory genes is still lacking.

**Methods:** We used univariate Cox model and Kaplan-Meier analysis to generate the forest plot of OS, PFI, DSS and identified the correlation between the altered expression of m7G regulators and patient survival in 33 cancer types from the TCGA and GTEx databases. Then, the “estimate” R-package, ssGSEA and CIBERSORT were used to depict the pan-cancer immune landscape. Through Spearman’s correlation test, we analyzed the correlation between m7G regulators and the tumor microenvironment (TME), immune subtype, and drug sensitivity of the tumors, which was further validated in NSCLC. We also assessed the changes in the expression of m7G related regulatory genes in NSCLC with regards to the genetic and transcriptional aspects and evaluated the correlation of METTL1 and WDR4 expression with TMB, MSI and immunotherapy in pan-cancer.

**Results:** High expression of most of the m7G regulators was significantly associated with worse prognosis. Correlation analyses revealed that the expression of majority of the m7G regulators was correlated with tumor immune infiltration and tumor stem cell scores. Drug sensitivity analysis showed that the expression of *CYFP1,2* was closely related to drug sensitivity for various anticancer agents (*p* < 0.001). Analysis of the pan-cancer immune subtype revealed significant differences in the expression of m7G regulators between different immune subtypes (*p* < 0.001). Additionally, the types and proportions of mutations in METTL1 and WDR4 and their relevance to immunotherapy were further described.

**Conclusion:** Our study is the first to evaluate the correlation between the altered expression of m7G regulators and patient survival, the degree of immune infiltration, TME and drug sensitivity in pan-cancer datasets.

## Background

Similar to the epigenetic code formed by DNA and histone modifications, post-transcriptional modifications in RNA profoundly affect cellular functions and fate, often termed as “RNA epigenetics” or “epitranscriptome,” and have attracted wide attention in recent years (He, 2010). There are over 150 known RNA modifications, which include N6-methyladenosine (m6A), N7-methylguanosine (m7G), 5-methylcytidine (m5C), pseudouridine (Ψ), N6,2′-O-dimethyladenosine (m6Am), N1-methyladenosine (m1A), alternative polyadenylation (APA), 2′-O-methylated nucleotides (Nm), N4-acetylcytidine (ac4C), adenosine-to-inosine RNA editing (I), and cytidine-to-uridine RNA editing (U), all of which contribute to normal development and homeostasis and their dysregulation is known to cause various diseases including cancer (Boccaletto et al., 2022; Zhao et al., 2017b; Frye et al., 2018; Roundtree et al., 2017; Barbieri et al., 2017; Barbieri and Kouzarides, 2020). Previous studies have focused more on m6A, one of the most common modified forms in eukaryotic cells, which have been demonstrated to play a role in embryogenesis, neurogenesis, hematopoiesis, and tumorigenesis in some studies (Zhao et al., 2017a; Zhang et al., 2017; Barbieri et al., 2017; Xu et al., 2021a). However, in recent years, modifications in non-m6A forms have also attracted increasing attention. For example, Xu et al. (2021b) investigated the potential relationship between regulators of non-m6a RNA modification and the clinical characteristics, TME status and GBM subtypes, and identified that three non-m6a RNA modification patterns were associated with distinct biological pathways and clinical characteristics.

m7G is widespread in numerous RNA cap structures as well as in tRNA, rRNA, mRNA and miRNA, is known to affect all the stages of RNA processing including splicing, export, decay, and controls the mRNA translation (Galloway and Cowling, 2019; Enroth et al., 2019; Zhang et al., 2019a; Malbec et al., 2019; Pandolfini et al., 2019). m7G modification processes are evolutionarily conserved, which are necessary for normal development (Malbec et al., 2019). Recently, a growing number of studies have implicated an important role of m7G-related regulators in the onset and progression of a variety of cancers, which were reported to be closely associated with patient prognosis. For example, METTL1 mediates m7G modification on tRNA, drives oncogene transformation and tumor formation by upregulating the translation of specific mRNAs such as growth-promoting proteins (Orellana et al., 2021). EIF3D is a subunit of the EIF3 complex with Cap-binding activity (Lee et al., 2016). EIF3D is elevated in gallbladder cancer (GBC) and promotes the proliferation and migration of malignant tumor cells (Zhang et al., 2021). Currently, lung cancer is one of the most prevalent malignancies worldwide and the leading cause of cancer-related death (Sung et al., 2021; Siegel et al., 2022). Recent studies reporting the targeting of specific m7G-related regulators have shown that m7G and lung cancer were closely related. METTL1-WDR4 mediates tRNA modifications and tRNA-decoded codon usage, which promotes mRNA translation leading to the proliferation and invasion of lung cancer cells. Furthermore, the WDR4/PML axis is also overactive in lung cancer and promotes tumor progression in the immunosuppressive and pre-metastatic microenvironment (Ma et al., 2021; Wang et al., 2017). EIF4G1, a scaffolding protein that anchors eIF4E, eIF4A1, the eIF3 complex, etc., is selectively upregulated in tumors, which is associated with enhanced Cap-independent initiation, especially in LUAD. The dramatically elevated expression of EIF4G1 also positively correlates with the expression of different cell cycle-related proteins (Wu and Wagner, 2021).

In this study, we analyzed the m7G-related regulators in pan-cancer datasets, and further evaluated the correlation of the differentially expressed m7G-related regulators with patient survival. Next, we specifically mapped the immune landscape in each cancer and analyzed the correlation between the differentially expressed m7G regulators and the TME across different cancers and drug sensitivity. Our findings may provide further direction for future studies exploring novel mechanisms and therapeutic targets in cancer.

## Methods

### Data sources and pre-processing

The normal tissue samples from genotype tissue expression (GTEx) and TCGA-GDC pan-cancer data including the transcriptional data (FKPM values) from 33 cancer types, immunophenotype data, stemness scores (calculated by DNA methylation and RNA expression, respectively), clinical variables, and genetic and transcriptional changes in the m7G-related genes were downloaded from UCSC Xena (https://xenabrowser.net/). The transcriptional data were recalculated with a unifying pipeline. Abbreviations for the different types of cancer are listed in Supplementary Table S1. Five m7G regulator gene sets were obtained from the GSEA’s MSigDB Home database (http://www.gsea-msigdb.org/gsea/index.jsp) as shown in Supplementary Table S2. The transcriptional and clinical data from GSE78220 (Hugo et al., 2016) based on the GPL11154 platform for pre-treated melanoma patients was obtained from the GEO database (https://www.ncbi.nlm.nih.gov/geo/). The IMvigor210 trial is a single-arm phase II study of atezolizumab in patients with metastatic urothelial carcinoma (Mariathasan et al., 2018), data from the IMvigor210 trial were obtained using the IMvigor210CoreBiologies R package (http://research-pub.gene.com/IMvigor210CoreBiologies/). We also used http://cis.hku.hk/TISIDB/ (Ru et al., 2019) to analyze the correlation between the expression of METTL1 and WDR4 and the expression of immunoinhibitors across pan-cancer.

### Gene expression and drug sensitivity analysis

The gene symbol of transcriptional data was obtained from Ensembl (https://asia.ensembl.org/index.html). Based on previous studies, we selected 28 cancer types that had more than five contiguous normal specimens (after merging with GTEx) among the 33 cancer types in further comparative analysis (Yu et al., 2020; Zhang et al., 2020a). The “corrplot” R package was used to analyze the correlation between the m6A regulators and m7G regulators. The R-package “limma,” “ggplot2,” and “reshape2” were used to investigate the correlation between the m7G regulators and the immune subtypes. The immune score, stromal score, and estimate score of pan-cancers were generated using the “estimate” R-package. The gene expression and drug sensitivity data were obtained from the CellMiner database (https://discover.nci.nih.gov/cellminer/home.do) and were filtered by clinical trials or FDA approved standards. *p*-value < 0.05 was considered as statistically significant.

### Survival analyses

The prognosis for overall survival (OS), progression-free interval (PFI) and disease-specific survival (DSS) of pan-cancer patients were compared with the expression level of m7G-related genes. Kaplan–Meier curves were generated to display the differences in the OS of patients. The univariate Cox proportional hazards regression models’ analyses were obtained for different samples from each type of cancer by “survival” and “survminer” R package.

### Correlation of ssGSEA score with the proportions of immune cell subsets in pan-cancer

We first quantified the level of enrichment of m7g-related genes in each sample by scoring with single-sample gene-set enrichment analysis (ssGSEA) (Shen et al., 2019). The proportions of 22 human immune cell subsets were calculated by CIBERSORT (Newman et al., 2015). The ssGSEA score and immune cell proportions were used to evaluate the correlation between the activity of m7g-related genes and the immune cell infiltration level in pan-cancer dataset, and the “limma” R-package was used to construct the corresponding heatmap.

### Mutation and microsatellite instability analysis

The alteration in the expression of *METTL1* and *WDR4* in the TCGA pan-cancer database was examined using the cBioportal database (https://www.cbioportal.org/). The “maftools” R package was employed to generate the pan-cancer mutation annotation format for the data downloaded from the UCSC Xena. The tumor mutation burden (TMB) score for each patient within pan-cancer was calculated and analyzed for its correlation with *METTL1* and *WDR4* expression, respectively. Mutation and microsatellite instability analysis (MSI) is characterized by a wide range of microsatellite sequence polymorphisms due to mutations in DNA polymerase, which is used as an indicator of genetic instability for cancer detection (Li et al., 2020). The MSI score of *METTL1* and *WDR4* for each patient within pan-cancer was also investigated in the same way as the TMB score.

### Statistical analyses

The mixed-effects linear models were used to compare the gene expression between the pan-cancer samples and their adjacent normal samples. The heatmap was created to visualize the differential gene expression between the tumor and normal tissues across all cancer types, while box plots were used to show the gene expression in each cancer type. Spearman correlation was used to perform the correlation analysis included in this study. Wilcox test or Kruskal-Wallis test was used to compare the different groups depending on the number of comparisons. All statistical analyses were performed using the R version 4.1.3 and all the reported *p*-values were two-tailed and *p* < 0.05 was considered as statistically significant.

## Results

### Acquisition of M7G-related regulatory gene listing and expression in 33 TCGA pan-cancers

**FIGURE 1:**
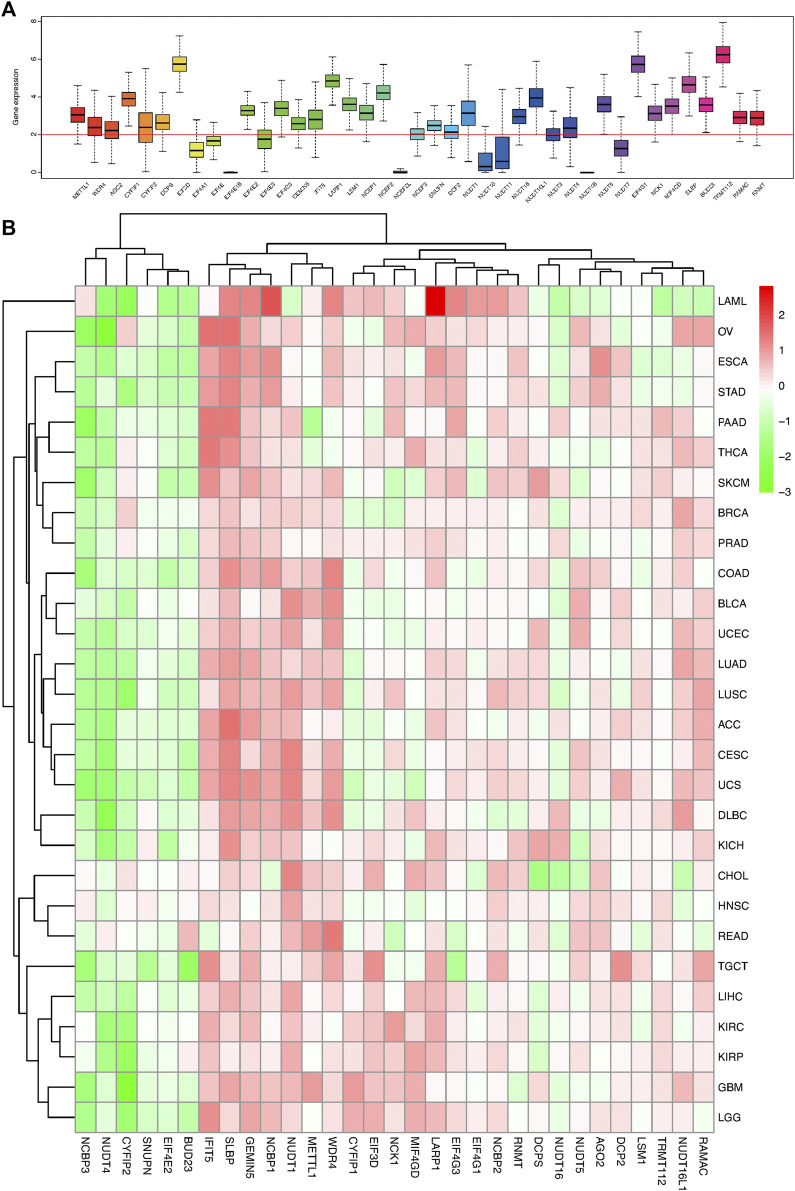
Box plot and heat map of the differential expression of 41 m7G regulators in 18 cancer types. **(A)** Box plot showing the expression of 41 m7G regulators in 18 TCGA cancer tissues. **(B)** Heat map showing the differential expression of 31 m7G regulators across 18 cancer types.

We obtained 41 m7G-related regulatory genes by searching GSEA’s MSigDB Home database and classified them into three classes based on their molecular function and biological process: N7 methylation (*WDR4, BUD23, METTL1, TRMT112, RAMAC, RNMT*); CAP-dependent translational initiation (Lin et al., 2007; Van Der Kelen et al., 2009) (*EIF4G1, NCK1, MIF4GD, SLBP, EIF3D*); and CAP formation genes (*SNUPN, EIF4A1, EIF4E, NCBP2, CYFIP1, LARP1, IFIT5, EIF4E1B, GEMIN5, CYFIP2, AGO2, LSM1, DCPS, EIF4E3, NCBP2L, NCBP1, NCBP3, EIF3D, EIF4G3, EIF4E2, DCP2, NUDT1, NUDT10, NUDT11, NUDT16, NUDT16L1, NUDT3, NUDT4, NUDT4B, NUDT5, NUDT7*), respectively. After analyzing the differential expression of 41 genes in 33 tumors and their matched normal tissues, 10 genes (*EIF4A1, EIF4E, EIF4E1B, EIF4E3, NCBP2L, NUDT10, NUDT11, NUDT3, NUDT4B, NUDT7*) with no significant difference in their expression (logFC absolute value <2) were eliminated. The remaining 31 genes were subjected to further pan-cancer analyses (Figure 1A).

The m7G related gene expression levels in pan-caner and normal samples were mapped in Figure 1B; Supplementary Figure S1. Most of the m7G regulators had a significantly higher expression in the tumor samples than the normal tissues, especially the N7 methylation genes. Correspondingly, there were some genes that were part of the CAP formation genes, such as *NCBP3, NUDT4, CYFIP2, SNUPN*, and *EIF4E2*, which were weakly expressed in most tumor tissues and highly expressed in normal tissues (Figure 1B; Supplementary Figure S1). Moreover, the expression of most of the CAP-dependent translational initiation genes was significantly increased in the tumor cells.

### Pan-cancer survival analysis of m7G-related regulators

**FIGURE 2:**
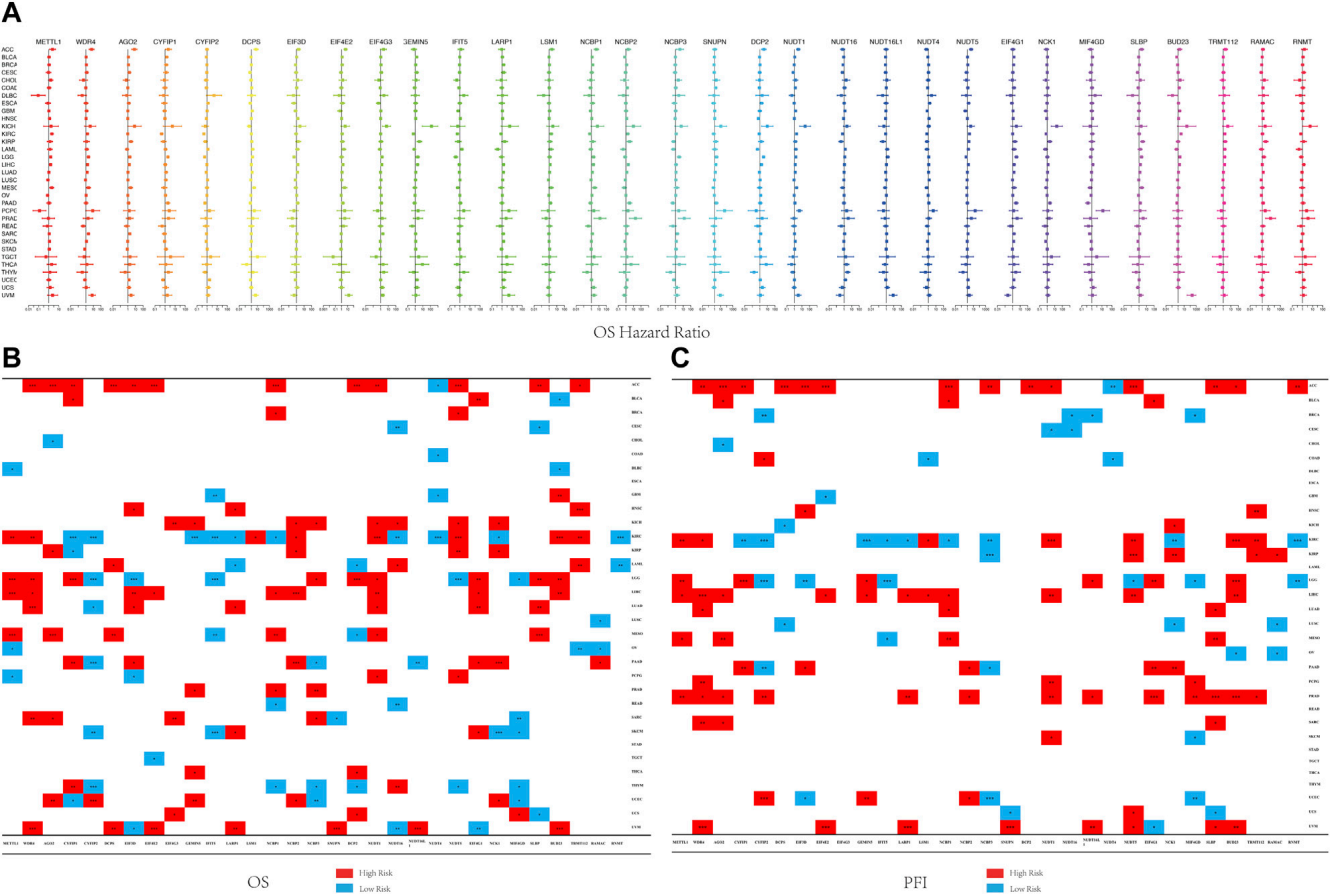
Forest map and survival analysis of m7G regulators in multiple cancers. **(A)** The forest map showing the overall survival risk ratio of 31 m6A-related genes across 33 TCGA cancer types. **(B)** Analysis of the correlation of m7G-related regulators with OS across multiple cancer types. **(C)** Analysis of the correlation of m7G-related regulators with PFI across multiple cancer types (*p < 0.05,**p < 0.01, and ***p < 0.001).

Previous studies have reported that m7G-related regulators, in particular, *METTL1* and *WDR4*, were associated with the prognosis of cancer patients (Tian et al., 2019; Zeng et al., 2021). However, only a few studies evaluated the influence of other m7G regulators on patient prognosis. For survival analysis, all 33 cancer types were tested with univariate Cox proportional hazards regression models, and *p*-value < 0.05 was considered as a statistically significant association. As shown in Figure 2A, *WDR4, NUDT1, BUD23* were high-risk factors in KIRC, LICH, GBM, and LGG patients. However, they were found to be low-risk factors in BLCA and DLBC patients. Kaplan-Meier survival curves showing the correlation between the 31 genes in the 33 cancers and OS, PFI and DSS are presented in Supplementary Figure S3. The results showed that the expression of the same gene in the same cancer did not result in significant differences in their OS, DSS, and PFI. Each m7G regulator was significantly associated with patient prognosis across multiple cancer types, and overall survival as the best indicator of endpoint could precisely differentiate the two risk groups. As shown in Figure 2, a general pattern was observed, wherein the elevated expression of a majority of the m7G regulators was associated with poorer survival and vice versa. For example, *METTL1* was highly expressed in most tumor tissues (Supplementary Figure S1), and a univariate Cox analysis showed that the high expression of *METTL1* was significantly associated with poor survival in cancer patients (Figure 2). Kaplan-Meier analysis in KIRC, MESO, and LGG patients clearly showed that increased expression of *METTL1* was significantly associated with a shorter OS, PFI and DSS (Figures 2B,C). However, the expression of *CYFIP2, IFIT5*, and *NUDT4* were negatively correlated with the prognosis of most cancer patients (Figure 2). The low expression of *CYFIP2* was associated with a significantly worse prognosis in KIRC, PAAD, and THYM patients (*p* < 0.001) (Figures 2B,C). The low expression of *IFIT5* was significantly associated with a poor prognosis in LGG, MESO, and SKCM patients (*p* < 0.001) (Figures 2B,C). Finally, the low expression of *NUDT4* was significantly associated with a worse prognosis in ACC, KIRC, and COAD patients (*p* < 0.001) (Figures 2B,C).

### Mapping the correlation between m7G-associated regulators and the tumor immune landscape and analysis of the tumor microenvironment in pan-cancer

**FIGURE 3:**
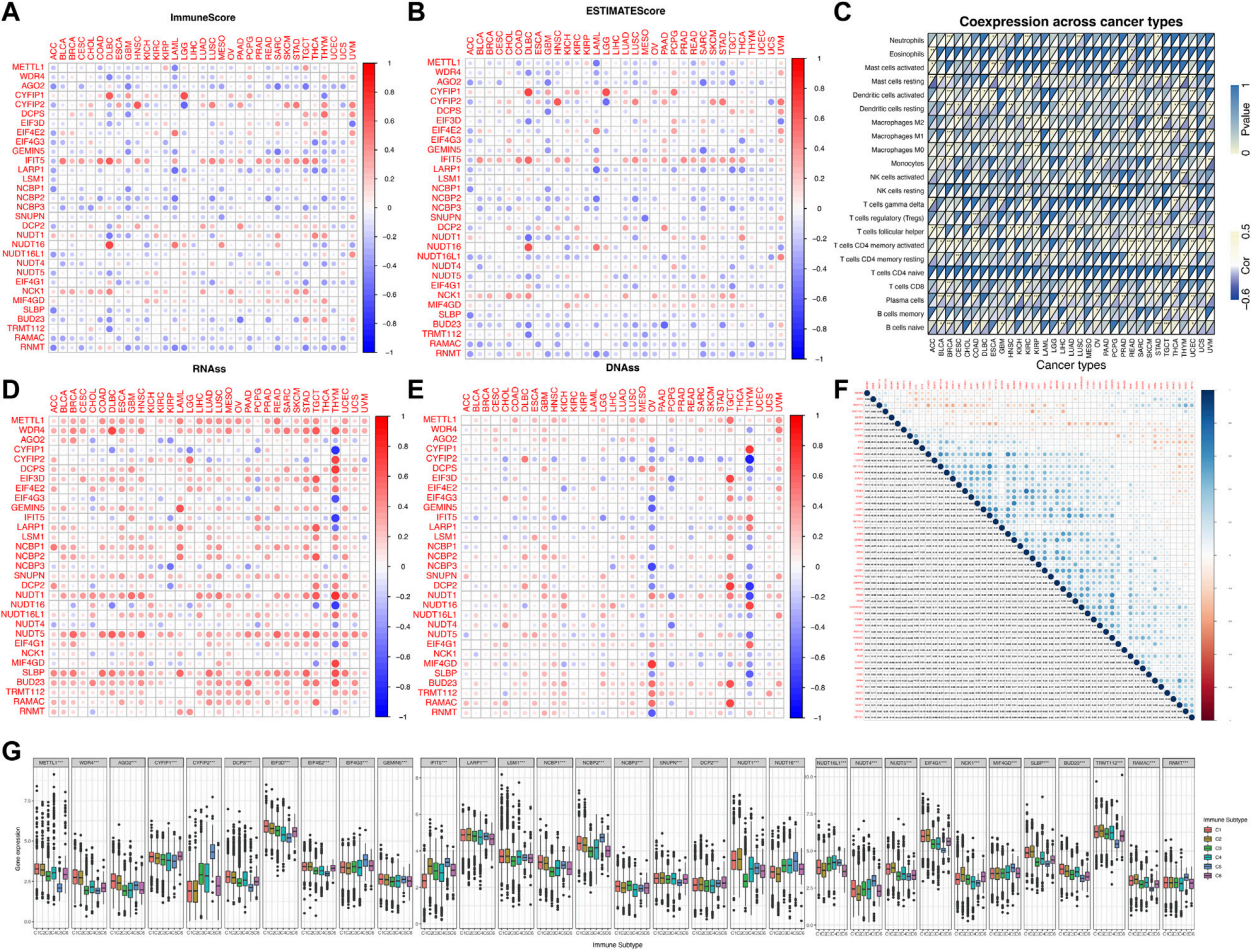
Correlation analysis between the expression of m7G-related regulators and the pan-cancer immune microenvironment with further analysis of m6A. **(A)** Correlation between the expression of 31 m7G methylation regulators and the immune score. **(B)** Correlation between the expression of 31 m7G methylation regulators and the stromal cell score. **(C)** Correlation between the enrichment level of m7G regulators (m7G geneset score) and tumor-associated immune cells in different cancers calculated by CIBERSORT. **(D)** Correlation between the expression of 31 m7G regulators and RNAss. **(E)** Correlation between the expression of 31 m7G regulators and DNAss. **(F)** The blue and red dots indicate that the expression of the m7G methylation regulators is positive and negative in relation to m6A methylation regulators, respectively. **(G)** The expression of m7G regulators within different immune infiltrate subtypes across all cancer types. The expression of m7G regulators within different immune infiltrate subtypes across all cancer types.

As we can see from Figures 3A,B, the expression levels of most of the m7G regulators were negatively correlated with the immune score, stromal cell score and ESTIMATE score. For example, the expression of AGO2 gene was significantly negatively correlated with the immune score in SARC, implying that increased expression of AGO2 resulted in lower immune cell infiltration in SARC patients. However, the expression of IFIT5 and NCK1 were positively correlated with the immune score in most cancers. To explore the relationship between changes in the overall levels of m7G regulators and the tumor immune landscape, we used the ssGSEA score to quantify the activity or enrichment levels of the 31 m7g-related genes in the cancer samples (Supplementary Table S3) and calculated the correlation between ssGSEA score and the tumor immune infiltration. As shown in Figure 3C, m7G-related genes were correlated to tumor-associated immune cell infiltration in different cancers, and had cancer species specificity. Among them, the Mast cells resting, Macrophages M2, Dendritic cells resting, T cells CD4 memory resting, T cells CD8, Plasma cells, and B cells naïve drew our attention, which were negatively correlated with the enrichment levels of m7G-related genes in most cancer types. Additionally, we found that the expression of most of the m7G regulators were positively correlated with RNAss (RNA methylation based stemness score) and DNAss (DNA methylation based stemness score) in 33 TCGA cancer types (Figures 3D,E). Then we explored the two groups (m6A and m7G) of regulators using deconvolution algorithm and found a positive correlation between them as shown in Figure 3F. The m6A regulators were associated with the degree of immune cell infiltration in several cancer types (Li et al., 2021). We also found that the expression of the m7G methyltransferase WDR4, was positively correlated with the expression of some of the m6A methyltransferases such as METTL13, WTAP, RBM15, and RBM15B. But another m7G methyltransferase regulator TRMT112, was negatively correlated with the expression of all m6A methyltransferases. The correlation of the individual m7G regulators is also shown in Figure 3F, where many of them were positively correlated with each other. We found that WDR4 expression was positively correlated with the expression of most of the regulators except NUDT16L1, NUDT16, NUDT4, CYFIP2, and IFIT5, suggesting its important role in the formation of m7G formation relative to METTL1. In addition, as shown in Figure 3G, the expression of m7G-related genes in pan-cancer was significantly different in immune subtypes C1 (wound healing), C2 (IFN-γ dominant), C3 (inflammatory), C4 (lymphocyte depletion), C5 (immune quiet) and C6 (TGF-β dominant) (*p* < 0.001).

### Correlation analysis between the expression of m7G regulators and drug sensitivity

**FIGURE 4:**
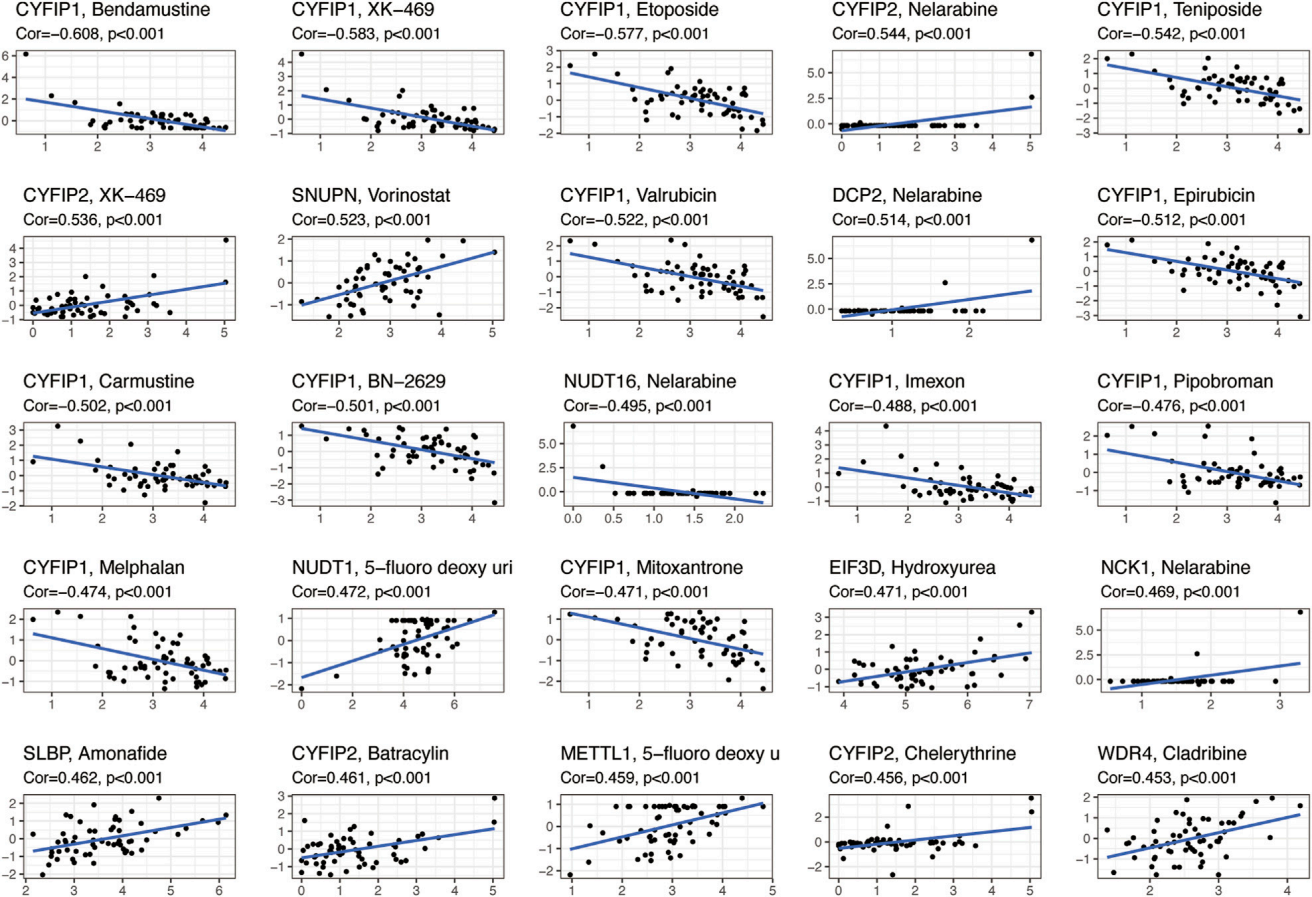
Scatterplots showing the association between the expression of m7G regulators and drug sensitivity (Z-score from CellMiner interface) using NCI-60 cell line data.

We analyzed the relationship between 31 regulatory m7G genes and drug sensitivity using the Cellminer database, and the top 25 drugs with significant correlation to their associated gene expression are presented in Figure 4. Of these, 16 drugs were significantly associated with the *CYFIP* gene family. *CYFIP1* expression was negatively correlated with drug sensitivity towards bendamustine, XK-469, etoposide, teniposide, valrubicin, epirubicin, carmustine, BN-2629, imexon, pipobroman, melphalan, and mitoxantrone. And the expression of *CYFIP2* was significantly positively correlated with the sensitivity towards nelarabine, XK-469, batracylin, and chelerythrine. The expression of *DCP2* and *NCK1* was positively correlated with drug sensitivity to nelarabine, while *NUDT16* expression was negatively correlated with sensitivity to nelarabine. Higher expression of *EIF3D* and *SLBP* was correlated with greater drug sensitivity to hydroxyurea and amonafide, respectively. Higher expression of *METTL1* and *WDR4* were correlated with greater drug sensitivity to 5-fluoro-deoxy-Uri and cladribine, respectively.

### Mutation patterns associated with m7G regulators and their clinical features in NSCLC

**FIGURE 5:**
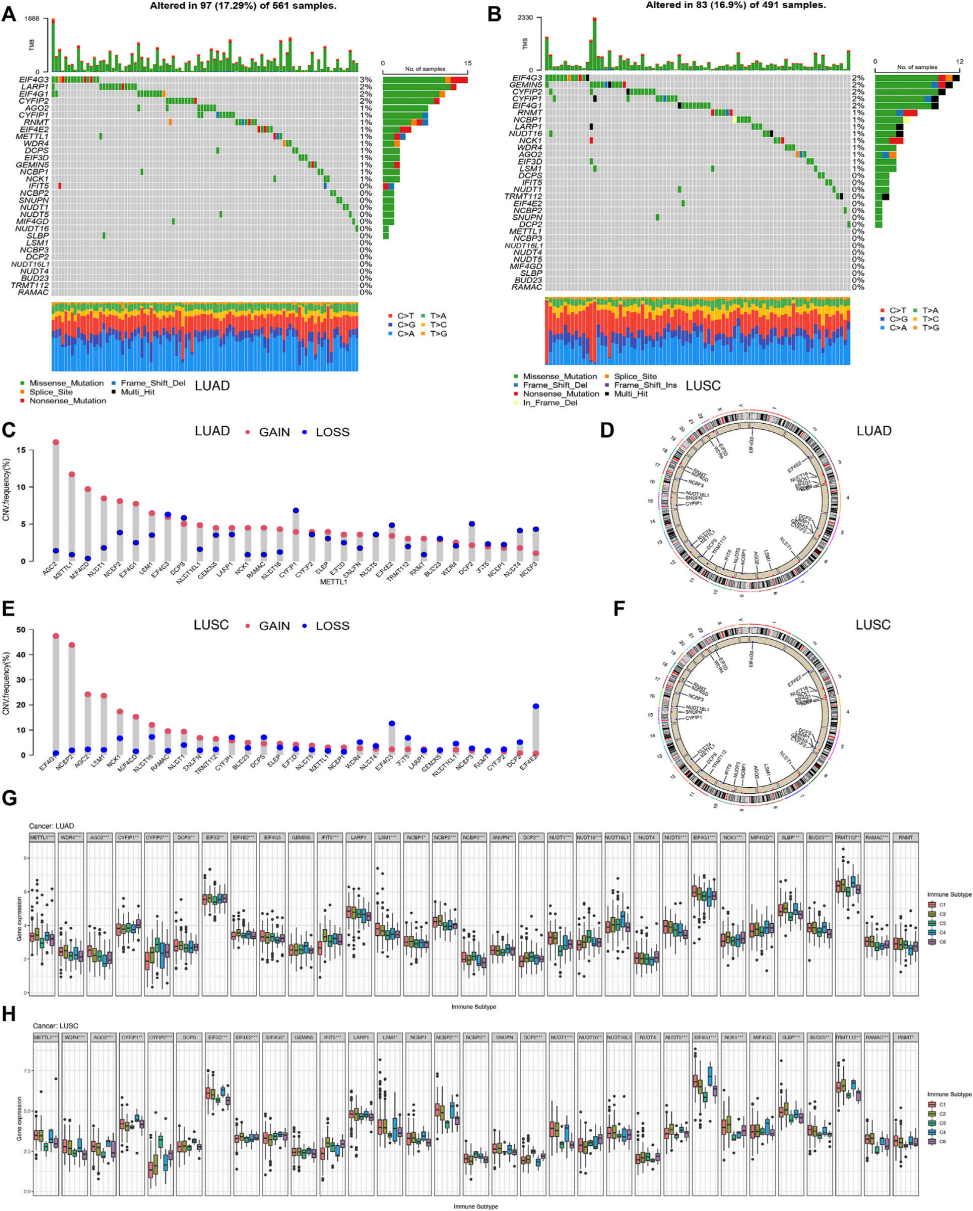
Correlation analysis between the expression of 31 m7G regulators and clinical features in LUAD and LUSC. **(A)** Mutation frequencies of 31 m7G regulators in 97 and 83 patients with LUAD. **(B)** Mutation frequencies of 31 m7G regulators in 97 and 83 patients with LUSC from the TCGA cohort. **(C)** Frequencies of CNV gain, loss, and non-CNV among m7G regulators in LUAD patients. **(D)** Chromosomal locations of CNV changes in m7G regulators in LUAD patients. **(E)** Frequencies of CNV gain, loss, and non-CNV among m7G regulators in LUSC. **(F)** Chromosomal locations of CNV changes in m7G regulators in LUSC patients. **(G)** Differences in the expression of m7G regulators across different immune subtypes of LUAD. **(H)** Differences in the expression ofm7G regulators across different immune subtypes of LUSC.

The pooled analysis of the incidence of somatic mutations in the above 31 m7G regulators showed a relatively high mutation frequency in both the LUAD and LUSC cohorts. Among the 561 LUAD samples, 97 samples (17.29%) had mutations in the m7G regulators (Figure 5A). Of these, *EIF4G3* had the highest mutation frequency (3%), followed by *LARP1* (2%), while the three N7 methylation genes, namely *BUD23, RAMAC,* and *TRMT112*, were not mutated. Among 491 LUSC samples, 83 samples (16.9%) had mutations in the m7G regulators (Figure 5B). *EIF4G3* also showed the highest mutation frequency (2%), followed by *GEMIN5* (2%). Similarly, in LUAD, three m7G methyltransferase genes, namely *BUD23, RAMAC,* and *METTL1*, had no mutations.

We then examined the somatic copy number changes and found predominant copy number changes in all the 31 m7G regulators. In the LUAD samples, copy number variants (CNV) were generally elevated in *AGO2, METTL1, MIF4GD, NCK1, RAMAC*, while it was generally lower in *CYFIP2, EIF4E2, DCP2, NUDT4, NCBP3* as shown in Figure 5C. While in the LUSC samples, CNV was generally elevated in *EIF4G1, NCBP2, AGO2, LSM1, MIF4GD*, and decreased in *EIF4G3, IFIT5, EIF4E2* as shown in Figure 5E. Figures 5D,F shows the CNV changes in m7G regulators and their position on the chromosomes in LUAD and LUSC samples, respectively. Figure 5G shows the expression of most of the m7G regulators except *EIF4G3, GEMIN5, LARP1, NUDT4*, and *RNMT,* in different immune types C1 (wound healing), C3 (inflammation), C2 (IFN-gamma dominant), C4 (lymphocyte depletion), and C6 (TGF-beta dominant) in LUAD samples. Furthermore, the clinical subgroup analysis in Supplementary Figure S4A showed significant differences in the expression of *CYFIP2, EIF3D, DCP2, NUDT16, SLBP*, and *RNMT* in pathologic T-stage LUAD. The expression of *METTL1, WDR4, NCBP3, DCP2, NUDT1, EIF4G1, BUD23*, and *RNMT* in LUAD was significantly different in the pathological stage N. The expression of *EIF3D* in LUAD was significantly different in the pathological stage M0/M1. Figure 5H shows the expression of m7G regulators in residues in the immune types C1 (wound healing), C3 (inflammation) LUSC, excluding the eight genes, namely*, DCPS, GEMIN5, LARP1, NCBP1, SNUPN, NUDT16L, NUDT4, and MIF4GD.* Furthermore, the clinical subgroup analysis displayed in Supplementary Figure S4B showed that the expression of *AGO2, CYFIP2, NUDT1, NUDT16,* and *NUDT4* in LUSC was significantly different in the pathologic T phase. The expression of *CYFIP1, GEMIN5, LARP1, NVBP1, NCBP3, NUDT16L1,* and *BUD23* in LUSC was significantly different in the pathological stage N. The expression of *GEMIN5, DCP2,* and *RNMT* in LUSC was significantly different in the pathological stage M0/M1.

Supplementary Figure S5A shows patients with high expression of WDR4, BUD23, METTL1, TRMT112, RAMAC, RNMT, EIF4G1, NCK1, MIF4GD, SLBP, EIF3D, SNUPN, NCBP2, LARP1, IFIT5, GEMIN5, AGO2, LSM1, DCPS, and NCBP1, with the exception of four negatively correlated genes (CYFIP1, CYFIP2, DCP2, NUDT16) and five unrelated genes (EIF4G3, IFIT5, NCBP3, NUDT16L1, NUDT4, MIF4GD). METTL1, AGO2, EIF4G3, NCBP2, NCBP3, NUDT1, NUDT16, NUDT4, NUDT5, EIF4G1, SLBP, BUD23, TRMT112, RAMAC, and RNMT were positively correlated with DNAss (*p* < 0, 05) in LUAD, while the expression of CYFIP1, CYFIP2, EIF4E2, GEMIN5, and IFIT5 in LUAD patients was negatively correlated with their DNAss score (*p* < 0.05). As shown in Supplementary Figure S5B, the expression of AGO2, WDR4, BUD23, METTL1, TRMT112, RAMAC, RNMT, SLBP, MIF4GD, EIF4G1, NUDT1, NUDT16L1, NUDT5, DCPS, EIF3D, GEMIN5, LARP1, LSM1, NCBP1, NCBP2, and SNUPN in LUSC patients was significantly positively associated with the RNAss score in LUSC (*p* < 0.05), with the exception of four negatively correlated genes (CYFIP2, EIF4G3, IFIT5, NUDT4) and six uncorrelated genes (CYFIP1, EIF4E2, NCBP3, DCP2, NUDT16, NCK1). Expression of WDR4, BUD23, METTL1, TRMT112, RAMAC, RNMT, AGO2, EIF3D, LSM1, NCBP2, NCBP3, SNUPN, NUDT1, NUDT16L1, EIF4G1, and MIF4GD were positively correlated with DNAss scores (*p* < 0.05) in LUSC, while the expression of CYFIP2, IFIT5, LARP1, and DCP2 were negatively correlated with the DNAss score in LUSC patients (*p* < 0.05). The expression of CYFIP1, CYFIP2, IFIT5, DCP2, NUDT4, and NCK1 in LUAD patients correlated positively with LUAD microenvironment scores (*p* < 0.05). However, the expression of BUD23, METTL1, TRMT112, RAMAC, SLBP, WDR4, DCPS, EIF3D, LARP1, LSM1, NCBP2, NCBP3, SNUPN, NUDT1, NUDT16L1, NUDT5, and EIF4G1 were negatively correlated with the micro-environmental assessment scores in LUAD patients (*p* < 0.05) (Figure 5A). Expression of WDR4, BUD23, METTL1, TRMT112, RAMAC, RNMT, SLBP, MIF4GD, EIF4G1, NUDT5, NUDT16L1, NUDT1, SNUPN, NCBP1, NCBP2, NCBP3, LSM1, GEMIN5, EIF3D, and AGO2 were negatively associated with the microenvironment score (*p* < 0.05) in LUSC, while the expression of CYFIP2, EIF4E2, IFIT5, DCP2, and NUDT4 were positively associated with the immune score, stromal score and estimation score in the LUSC samples (*p* < 0.05) (Supplementary Figure S5B).

### METTL1 and WDR4 play a vital role in tumorigenesis of pan-cancer

**FIGURE 6:**
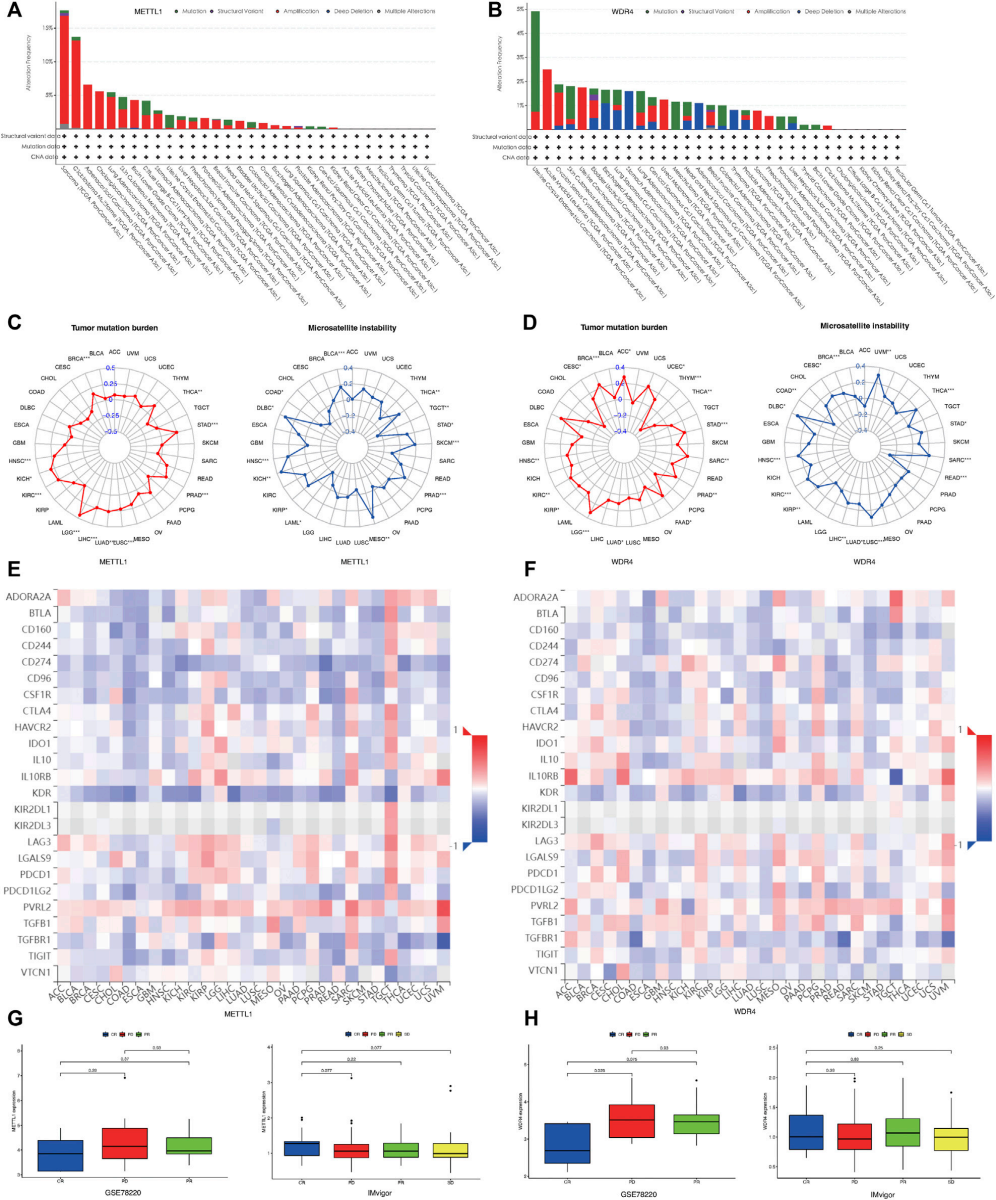
Mutational landscape of METTL1 and WDR4 in cancer. **(A)** METTL1 mutation frequency in multiple TCGA pan-cancer studies according to the cBioPortal database. **(B)** WDR4 mutation frequency in several TCGA pan-cancer studies according to the cBioPortal database. **(C)** Radar plot visualizing the relationship between METTL1 expression and TMB. Radar plot visualizing the relationship between WDR4 expression and TMB. **(D)** Radar plot visualizing the relationship between METTL1 expression and MSI. Radar chart visualizing the relationship between WDR4 expression and MSI. **(E)** The relationship between METTL1 expression and immunoinhibitors in pan-cancer. **(F)** The relationship between WDR4 expression and immunoinhibitors in pan-cancer. **(G)** The expression levels of METTL1 were significantly different in four groups of IMvigor rather than GSE78220. **(H)** The expression levels of WDR4 were significantly different in three groups of GSE78220 rather than IMvigor.

Using the cBioportal database, we examined the pan-cancer changes in *METTL1* and *WDR4* in the TCGA database. As shown in Figure 6A, the results reflected that the highest frequency of change in *METTL1* was approximately 18% in sarcoma, while 5.5% in LUAD and 1% in LUSC. Among the different types of genetic alterations, amplification was the most common. Figure 6B shows that the highest alteration frequency in *WDR4* was in endometrial carcinoma of the uterine body, about 4.9%, while it was 1.8% in LUAD and 1.82% in LUSC. Gene mutation was the most common type of alteration. The TMB radar plot of *METTL1* in Figure 6C shows the significant positive correlation of METTL1 mutations with STAD, PRAD, LUSC, LUAD, LIHC, LGG, KIRC, KICH, HNSC, and BRCA, while there was a significant negative correlation with THCA. The METTL1-MSI radar plot in Figure 6D shows the significant positive correlation of MSI with STAD, THCA, PRAD, MESO, KIRP, KICH, HNSC, and BRCA, while there was a negative correlation with THCA. The TMB radar plot of WDR4 in Figure 6E shows significant positive correlation of *WDR4* mutation with STAD, PRAD, LUSC, LUAD, LIHC, LGG, KIRC, KICH, HNSC, and BRCA, while there was a significant negative correlation with THCA. The WDR4-MSI radar plot in Figure 6F shows significant positive correlations of MSI with UVM, STAD, THCA, SARC, READ PRAD, MESO, KIRP, KICH, HNSC, DLBC, and BRCA, while there was negative correlation with READ and COAD.

To investigate the relationship between METTL1/WDR4 expression and immunotherapy response in pan-cancer, we first explored the correlation between the expression of METTL1 and WDR4 with the expression of immunoinhibitors in the TISIDB database (Figures 6E,F). CD274 programmed death protein ligand 1 (PD-L1, CD274, B7-H1) has been shown to play a role in the regulation of immune responses and peripheral tolerance, and play a critical role in the induction and maintenance of tolerance to autoimmunity (Fabrizio et al., 2018; Huang et al., 2021). As shown in Figures 6E,F, the expression levels of *METTL1, WDR4*, and *CD274* was inconsistent across different tumors. *METTL1* showed a negative correlation while *WDR4* was more positively correlated overall. We next chose the IMvigor210 and GSE78220 cohort to explore the association of *METTL1* and *WDR4* levels with the outcome of different immunotherapy. In GSE78220, the clinical outcomes were divided into CR (Complete response), PR (Partial response), and PD (Progressive disease), and the expression level of *WDR4* was statistically significant in the different groups. A lower expression level of *WDR4* was associated with a positive immunotherapy response (*p* = 0.026) in Figure 6G. However, in the clinical groups in Imvigor, which were divided into the following four groups: CR, PD, PR, and SD (Stable disease), a higher expression level of METTL1 was associated with a positive immunotherapy response (*p* = 0.077) in Figure 6H. These results were consistent with the above findings.

## Discussion

Previously, m7G was thought to be a representative of the CAP structure at the 5′ end of most mammalian mRNAs, which kept it stable during genetic inheritance (Furuichi, 2015). Our study evaluated the differential expression of the m7G regulators and their association with the immune landscape, including the TME, immune subtypes, and stem cell scores in 33 cancer types from the TCGA pan-cancer dataset. Then, the findings were validated in NSCLC. The expression of the m7G regulators in the tumor and normal tissues were significantly different as shown in Supplementary Figure S1, which suggest the potential role of these differentially expressed genes in tumorigenesis. Many CAP-dependent translational initiation genes were associated with transcription initiation of mRNA, which reflects the high transcription requirements of tumor tissues.

m6A, a widely studied form of RNA methylation modification, is essential for regulating RNA transcription, processing and translation, which in turn affects cellular metabolic activities. Different m6A modification patterns are known to interact with the immune phenotypes of tumors, including immune rejection, immune activation, and immune inertness, thus, estimating the m6A pattern of a tumor might enable the characterization of TME infiltration and provide guidance for improved immunotherapy strategies (Du et al., 2021; Zhang et al., 2020b). In the present study, we analyzed the correlation between m6A and m7G regulators using a deconvolution algorithm and found that most of the genes in the two groups showed a positive correlation with each other. This suggests that some common factors between the two groups may work together in one pathway. For example, the altered expression of *TRMT112* could affect the tumor suppressive role of *WBSCR22* in pancreatic cancer (PC), leading to tumor evolution (Khan et al., 2022). van Tran et al. (2019) identified METTL5 as the enzyme responsible for 18S rRNA m6A modification and showed that it formed a heterodimeric complex with the known methyltransferase activator TRMT112, in order to be metabolically stable in cells. Therefore, we hypothesized that identifying and blocking some common regulators in both the methylation modification pathways might be more effective than individually blocking some of the key genes.

We next examined the expression of m7G regulators in different immune-infiltrating subtypes of TME and their correlation with different immune subtypes. Specifically, *METTL1, WDR4, CYFIP2, NUDT5, EIF4G1, NCK1, MIF4GD, SLBP, IFIT5*, and *NUDT1* were found to be significantly associated with the immune subtypes (*p* ≤ 0.01). *METTL1, WDR4, EIF3D*, and *NUDT1* were found to be associated with the more aggressive immune subtypes C1 and C2. However, *CYFIP1* was significantly associated with the C6 subtype. We speculated that the increased expression of *CYFIP1, METTL1, WDR4, EIF3D*, and *NUDT1* predicted poor prognosis, and *CYFIP1* had stronger correlation to the TGF-β immune signature, and higher lymphocytic infiltration than the other regulators, with an even distribution of type I and type II T cells. Moreover, *NCBP3* showed increased expression in the immune subtype C3, which indicated a better prognosis and longer survival, suggesting that it may have an inhibitory role in cancers. However, *CYFIP2*, *EIF4G3, IFIT15, NCBP2, NUDT16, NUDT4*, and *MIF4GD* showed the highest expression in the immunologically quiescent C5 subtype, which was associated with a better prognosis.

The interaction between tumor cells and stromal components forms a functionally complex TME (Schaaf et al., 2018; Hinshaw and Shevde, 2019). Cancer-associated fibroblasts (CAFs) are mainly distributed around blood vessels or in the fibrous interstitium around tumors, and secrete cytokines, ECM components and related enzyme molecules. CAFs expressing the fibroblast activation protein induces a pro-inflammatory and pro-angiogenic microenvironment, promotes proliferation and increases stemness characteristics in a variety of cancer cells (Mao et al., 2021; Peltier et al., 2022). There are a variety of immune infiltrating cells in the TME, among which CD8^+^ or cytotoxic T lymphocytes (CTL) play a tumor-killing function. Wu et al. (2022) demonstrated that NELFB in CD8^+^ T lymphocytes played an important role in antitumor immunity associated with TCF1, promoting TCF1-bound transcriptional enhancers and promoter chromatin accessibility. Generally, M1-type macrophages play pro-inflammatory and anti-tumor functions, but tumor-associated macrophages (TAMs) in the TME are M2-type. Xu et al. (2021a) elucidated the mechanism by which hypoxia and glioma affected autophagy and M2-like macrophage polarization through exosomes, thereby promoting the formation of an immunosuppressive microenvironment.

According to the immune estimation algorithm, m7G regulators also correlated differentially with immune cell infiltration in the TCGA 33 Pan-cancer atlas. We found that the m7G-related genes were mainly negatively correlated with the stroma scores (Supplementary Figure S3), of which CAFs were the major players. Since the expression of most of the m7G regulators were negatively associated with immune scores in the TCGA 33 Pan Cancer Atlas, we next investigated the correlation between the overall expression level and immune cells. After calculating the ssGSEA score representing the activity of 31 m7G-regulated genes in the pan-cancer cohort, we found that the ssGSEA score was negatively correlated with higher infiltration of some immune cells especially Mast cells, T cells CD4 memory resting, T cells CD8 and B cells, these cell types have been shown by multiple studies to hinder tumor progression and promote a hyperimmune state of the tumor microenvironment and affect patient outcomes (Lichterman and Reddy, 2021; Lohr et al., 2013; Zhang et al., 2019b; Fu and Jiang, 2018; Wouters and Nelson, 2018). These results suggested that in future studies, one must consider linking changes in individual genes and changes in overall regulatory genes to analyze the possible effects of m7G methylation, which may lead to more realistic results.

In general, we found that the m7G-related gene expressions were correlated with the levels of immune or stromal infiltration, tumor purity, and prognosis. A previous study reported that in T cell acute lymphoblastic leukemia, the epigenetic loss of NUDT16 mediated the activation of C-MYC and promoted tumor progression (Anadon et al., 2017). Notably, certain genes that were positively associated with immune infiltration were also involved in tumor progression. For example, *IFIT5* was shown to promote tumor cell invasion and migration by inducing EMT and downregulating miR-99a in bladder cancer (Huang et al., 2019). Therefore, the selection of specific genes as immune-related markers with specific molecular functions should be carried out and tested in tumor signaling pathways in order not to isolate oneself from reality.

Cancer stem cells (CSC) promote cancer progression due to their ability to self-renew and to invade and mediate therapeutic resistance (Schonberg et al., 2014). The expression of most genes was positively associated with both DNAss and RNAss scores. But *IFIT5* and *CYFIP2* were significantly negatively correlated with DNAss in some tumors. Moreover, the dysregulated expression of m7G regulators were generally significantly associated with OS, DSS and PFI in patients from the TCGA dataset in this study (Supplementary Figure S3). Obviously, the prognosis of patients with the same tumor correlated with the expression of multiple regulators. The Cellminer drug sensitivity analysis confirmed the significant correlation of almost all m7G regulators with sensitivity towards several of the currently used anti-cancer agents. CYFIP1 is one of the components of the CYFIP1-EIF4E-FMR1 complex, which enables its binding to the mRNA cap, mediating translational repression (Santini et al., 2017). Elevated CYFIP1 expression is known to suppress the drug sensitivity to bendamustine, XK-469, etoposide, teniposide, valrubicin, epirubicin, imexon, pipobroman, melphalan, and mitoxantrone. However, CYFIP1 expression has been negatively correlated with patient prognosis in several cancer types (KIRC, KIRP, UCEC), suggesting the tumor repressive role of CYFIP1. Oguro-Ando et al. (2015) showed that the overexpression of CYFIP1 in mammals dysregulated the mTOR signaling pathway. After treatment with rapamycin, the morphological abnormalities of neurons arising due to CYFIP1 overexpression were rescued in mice. Quantitative studies have shown that patients with renal tumors (ccRCC) benefitted from treatment with mTOR inhibitors such as everolimus and temsirolimus (Voss et al., 2014; Dong et al., 2019). CYFIP1 overexpression may play an important role in cancers with a predominantly altered mTOR signaling pathway.

Recent studies have mainly focused on the METTL1 and WDR4 complex, which are known to play important role in the modification of tRNAs (Dai et al., 2021). METTL1 has been reported to be highly expressed in a variety of cancers and was associated with tumor initiation, metastasis and chemo-sensitivity. Moreover, some studies have shown that METTL1 was associated with the immunosuppressive tumor microenvironment and stemness indices, and that its expression reflected the sensitivity of immune checkpoint blockade (ICB) therapy (Ma et al., 2021; Tian et al., 2019; Dai et al., 2021; Liu et al., 2019; Gao et al., 2022). WDR4 is also involved in a variety of cellular functions, including signal transduction, cell cycle promotion, and apoptotic cell death process (Lin et al., 2018; Michaud et al., 2000). Some studies showed that the aberrant expression of WDR4 was observed in various malignant cancers and was significantly associated with the overall survival outcomes. The expression level of WDR4 is also strongly associated with tumor immunity, such as immune scores and tumor-infiltrating immune cells (Zeng et al., 2021). In our study, we found that the expression levels of METTL1 and WDR4 were correlated with immunoinhibitors in pan-cancer datasets. Using data sets from previous studies (IMvigor210 and GSE78220 cohort), we further verified that the expression levels of METTL1 and WDR4 were related to the efficacy of immunotherapy. TMB is a valid predictive marker for tumor immunotherapy, effectively identifies and distinguishes individuals that could benefit from immunotherapy (Chan et al., 2019). Our TMB and MSI analyses for *METTL1* and *WDR4* reflected the mutation frequency and proportion at the molecular level in different tumors and showed the correlation between mutation load and tumor progression. *METTL1* could serve as a predictive biomarker for immunotherapy efficacy in BRCA, STAD, PRAD, and LUSC, while WDR4 had similar role in BRCA, THYM, STAD, and LGG. High mutation rates of *METTL1* and *WDR4* showed significant correlations in certain tumors, and the combination of the two biomarkers could be used to construct a precise model for predicting tumor progression and response to immunotherapy in BRCA, STAD, and LGG.

By examining the m7G regulators in NSCLC, we observed that the expression of these genes varied in different immune subtypes within the same cancer. Thus, we hypothesized that the tumor suppressive or promoting role of these genes were subtype-specific and subsequently confirmed it in further clinical subgroup analyses. In our study, high expression of *WDR4, EIF4G1* and *SLBP* were significantly associated with a poor OS (*p* < 0.001) and increased tumor stem cell score in LUAD. Meanwhile, they were associated with lower immune scores, confirming the negative impact of their increased expression on patient survival. Similarly, in LUSC, higher expression of *AGO2, EIF3D*, and *LSM1* were positively correlated with tumor stem cell score but negatively correlated with immune infiltration degrees, indicating a poor prognosis. TMB and CNV analysis were performed to investigate the possible mechanisms underlying the altered expression of the m7G regulators. We found that the TMB of m7G regulators was low in both LUAD and LUSC patients, and the predominant alteration was mutation followed by gene amplification. However, in terms of CNV, LUAD, and LUSC had significant different alterations. Through the combination of TMB and clinical subgroup analysis, we concluded that CNV was not the sole factor influencing the expression of m7G regulators in both LUAD and in LUSC.

## Conclusion

In summary, based on our systematic analyses of m7G regulators, we demonstrated the correlation between the differential expression of m7G regulators and patient survival, cancer immune landscape and tumor microenvironment, and concluded that the altered expression of *WDR4, METTTL1, NUDT1, IFIT5*, and *CYFIP2* were associated with poor prognosis in cancer patients. Excluding *NCBP3, NUDT4, CYFIP2, SNUPN, EIF4E2*, and *BUD23*, most m7G regulators were upregulated in the primary tumors, all of which were associated with at least two of the following traits: more aggressive immune subtype; lower degree of immune infiltration; and a poor survival. The increased expression of *WDR4, METTTL1, NUDT1*, and *CYFIP2* enhanced the sensitivity of some anti-cancer drugs such as hydroxyurea, L-asparaginase, 5-fluoro-deoxy-uridine 10mer, and chelerythrine. Upregulated expression of the above genes was often associated with a poor prognosis, their increased expression seemed to improve the sensitivity of patients to specific drugs. The expression levels of *METTL1* and *WDR4* was associated with immunotherapy and they could serve as potential prognostic markers. Furthermore, an individual analysis of NSCLC patients revealed that multiple genes (validated *METTTL1* and *WDR4*, as well as *EIF4G1, SLBP, AGB2, EIF3D*, and *LSM1*) might play important roles in NSCLC progression, which could serve as potential biomarkers for predicting patient prognosis and response to immunotherapy. However, the oncogenic or tumor suppressing role of the individual m7G regulators depends on their specific molecular functions, thus it would be necessary to investigate their individual functional role in different types of cancer.

## Data Availability

Publicly available datasets were analyzed in this study. Thenames of the repository/repositories and accession number(s) can be found in the article/Supplementary Material.

## Supplementary material

SUPPLEMENTARY FIGURE S1 Boxplot of m7G regulators differential expression between cancer and adjacent normal tissues. The blue boxplots indicate the normal tissues. The red boxplots indicate the cancer tissues.

SUPPLEMENTARY FIGURE S2 The forest map of PFI and DSS in 31 m7G-related genes across 33 TCGA cancer types. **(A)** The forest map of PFS hazard ratio of 31 m7G-related genes across 33 TCGA cancer types. **(B)** The forest map of DSS hazard ratio of 31 m7G-related genes across 33 TCGA cancer types. **(C)** Survival correlation analysis of m7G related regulators in DSS across multiple cancer types. **(D)** The correlation relationship among the expression of 31 m7G methylation regulators and tumor purity. **(E)** Correlation between the expression of 31 m7G methylation regulators and the stromal cell score.

SUPPLEMENTARY FIGURE S3 Survival analysis of m7G related regulators across multiple cancer types. The red line in the photos indicates high expression and the blue line in the photos indicates low expression.

SUPPLEMENTARY FIGURE S4 Correlation analysis between 31 m7G regulators expression and clinical characteristics in LUAD and LUSC, respectively.

SUPPLEMENTARY FIGURE S5 Correlation analysis of m7G regulators and LUAD, LUSC microenvironment, and stem cell scores.**(A)** The correlation relationship between m7G methylation regulators expression and immune score, stromal cell score, stem cell score and immune microenvironment in LUAD. **(B)** The correlation relationship between m7G methylation regulators expression and immune score, stromal cell score, stem cell score, and immune microenvironment in LUSC.
