# Supplementary figures and images for: Integrative pan-cancer analysis and clinical characterization of the N7-methylguanosine (m7G) RNA modification regulators in human cancers
Source: Front Genet. 2022 Sep 26;13:998147. doi: 10.3389/fgene.2022.998147 (PMC9549978; doi:10.3389/fgene.2022.998147)

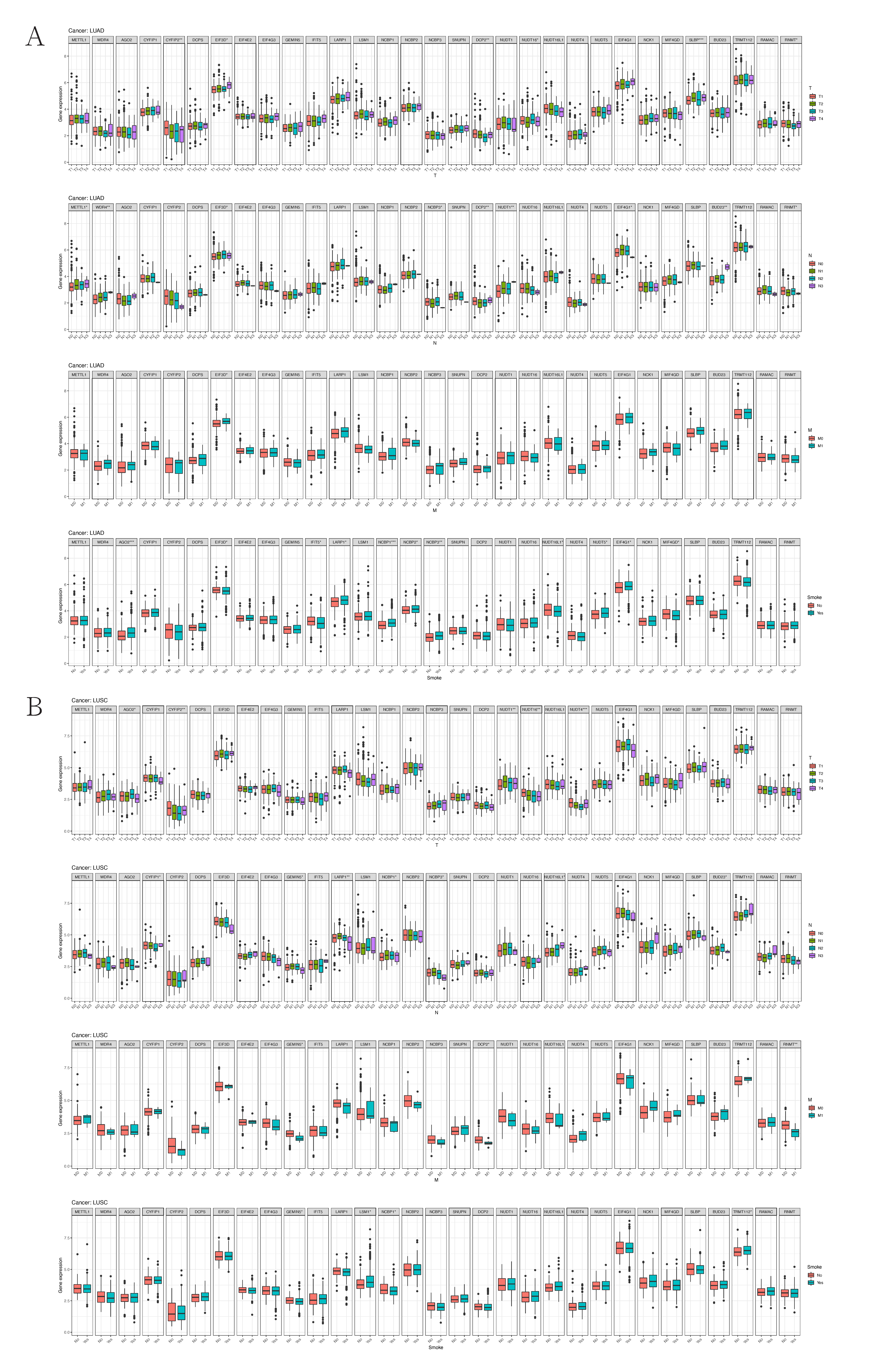

Supplement: Supplementary file 2 [file Image4.tif]
